# Associations of metabolic dysfunction-associated fatty liver disease and hepatic fibrosis with bone mineral density and risk of osteopenia/osteoporosis in T2DM patients

**DOI:** 10.3389/fendo.2023.1278505

**Published:** 2023-12-04

**Authors:** Wei Zhang, Yuhua Li, Shangjian Li, Jingqi Zhou, Kai Wang, Zhibin Li, Ning Chen, Xueqin Chen

**Affiliations:** ^1^ Xiamen Key Laboratory of Cardiac Electrophysiology, Xiamen Institute of Cardiovascular Diseases, The First Affiliated Hospital of Xiamen University, School of Medicine, Xiamen University, Xiamen, China; ^2^ School of Mechanics and Civil Engineering, China University of Mining and Technology-Beijing, Beijing, China; ^3^ Department of Endocrinology, Zhongshan Hospital (Xiamen), Fudan University, Xiamen, China; ^4^ Epidemiology Research Unit, Translational Medicine Research Center, The First Affiliated Hospital of Xiamen University, School of Medicine, Xiamen University, Xiamen, China; ^5^ Institute of Clinical Medicine, The First Affiliated Hospital of Xiamen University, School of Medicine, Xiamen University, Xiamen, China

**Keywords:** osteoporosis, osteopenia, diabetes, MAFLD, hepatic steatosis, hepatic fibrosis

## Abstract

**Background:**

Existing evidence on the associations of liver steatosis and fibrosis with bone mineral density (BMD) and risk of osteopenia/osteoporosis was limited with conflicting results. We aimed to evaluate the associations of metabolic dysfunction-associated fatty liver disease (MAFLD) and hepatic fibrosis with BMD and risk of osteopenia/osteoporosis in type 2 diabetes mellitus (T2DM) patients.

**Methods:**

Baseline information of an ongoing cohort of 249 T2DM patients in Xiamen, China was analyzed. MAFLD was defined as the presence of hepatic steatosis [diagnosed by either hepatic ultrasonography scanning or fatty liver index (FLI) score >60] for T2DM patients. BMD was measured using dual-energy x-ray absorptiometry at total lumbar (L2–4), femur neck (FN), and total hip (TH) and was categorized as normal (T ≥ −1.0), osteopenia (−2.5 < T < −1.0), or osteoporosis (T ≤ −2.5) according to its minimum T-score.

**Results:**

Among the 249 T2DM patients, prevalence rates of MAFLD, osteopenia, and osteoporosis were 57.8%, 50.6%, and 17.7%, respectively. Patients with MAFLD had significantly higher BMD T-scores of L2–4, FN, and TH and the minimum as well as lower prevalence of osteoporosis than patients without MAFLD. Hepatic steatosis indices, including FLI score, fatty liver (FLI ≥ 60 or hepatic ultrasonography scanning), and MAFLD, were significantly and positively associated with all T-scores, while hepatic fibrosis index and FIB-4 score, but not NAFLD fibrosis score (NFS), were negatively associated with all T-scores. MAFLD was significantly associated with the decreased risk of osteopenia/osteoporosis and osteoporosis with unadjusted odds ratios (ORs) (95% CI) of 0.565 (0.324–0.987) and 0.434 (0.224–0.843) (both *p*-values < 0.05), respectively. As for liver fibrosis, FIB-4 score, but not NFS, was significantly associated with elevated risk of osteoporosis with an unadjusted OR (95% CI) per SD increase of FIB-4 score of 1.446 (1.080–1.936, *p*-value = 0.013). Adjusting for potential confounding variables, especially body mass index, in the multivariable regression analyses, all associations of hepatic steatosis and fibrosis indices with BMD and risk of osteopenia/osteoporosis were not statistically significant.

**Conclusion:**

MAFLD and hepatic fibrosis were not significantly associated with BMD and risk of osteopenia/osteoporosis independent of obesity. Nevertheless, screening and management of MAFLD and osteopenia/osteoporosis were still important for the prevention of fracture in T2DM patients.

## 1 Introduction

Metabolic dysfunction-associated fatty liver disease (MAFLD) has been introduced and defined as patients with both hepatic steatosis and any of the three metabolic conditions—overweight/obesity, diabetes mellitus, or metabolic dysfunction—in lean populations, rather than the absence of alcohol abuse or other chronic liver diseases in 2020, and therefore, has been suggested as a replacement for non-alcoholic fatty liver disease (NAFLD) (1, 2). MAFLD affects approximately a third of the global population, and its related health burden has grown positively with increasing prevalence of type 2 diabetes mellitus (T2DM) and obesity (3, 4). Recently, metabolic dysfunction-associated steatotic liver disease (MASLD) has been proposed by a consensus group to replace the term NAFLD (5). Based on a cross-sectional analysis of the 2017–2020 cycle of National Health and Nutrition Examination Survey (NHANES) in the US population, the prevalence of steatotic liver disease (SLD) was 42.1%, and among them, 89.4%, 7.7%, 2.4%, 0.4%, and 0.1% were defined as MASLD, MetALD (MASLD + significant alcohol consumption), MASLD-viral hepatitis, alcoholic liver disease (ALD) (significant alcohol consumption without metabolic dysfunction), and cryptogenic, respectively (6). MAFLD shares insulin resistance and compensatory portal or systemic hyperinsulinemia with T2DM as common pathophysiological mechanisms; meanwhile, fat accumulation in liver and alterations in both energy metabolism and inflammatory signals are also involved in these two conditions (7, 8). Osteoporosis is characterized by the deterioration of micro-architecture in bone tissue and reduced bone mass, affects more than 200 million people globally, and has become a worldwide common chronic disease due to the significant economic burden of osteoporosis-related fracture (9, 10). T2DM patients have increased risks of fractures, possibly due to osteoporosis or insulin use (11, 12), although numerous studies found that bone mineral density (BMD) in T2DM patients was normal or increased compared to that of the age-matched controls (11, 13, 14).

Some previous studies reported that higher BMI protected the risk of osteoporosis in diabetes patients (15, 16). However, available evidence of associations between MAFLD and BMD or risks of osteoporosis was scarce. Three studies based on cross-sectional analyses of the 2017–2018 cycle of NHANES in the US population older than 50 years found similar results that liver steatosis and fibrosis are not independently associated with osteopenia or osteoporosis although patients with MAFLD showed increased BMD than their controls (17–19). However, a cross-sectional study from 1,872 obese individuals in Rome, Italy found that higher Fibrosis-4 (FIB-4) score, an index of liver fibrosis, was significantly associated with lower BMD and increased risk of osteopenia/osteoporosis (20). Yet, there was no evidence available about the associations of MAFLD and liver fibrosis with BMD and osteopenia/osteoporosis in patients with T2DM, although MAFLD and osteoporosis share with T2DM some common pathophysiological mechanisms, such as insulin resistance, hyperinsulinemia, pro-inflammatory state of liver and adipose, enhanced lipotoxicity, and excessive reactive oxidative stress (7, 8, 14). In the present study focusing on T2DM patients, firstly we aimed to investigate the associations of MAFLD and liver fibrosis indices with BMD. Secondly, we also aimed to test the associations of MAFLD and liver fibrosis indices with risks of osteopenia/osteoporosis and osteoporosis. Thirdly, we aimed to explore whether the associations of MAFLD and liver fibrosis with BMD and risk of osteopenia/osteoporosis were independent of general obesity.

## 2 Materials and methods

### 2.1 Study population

This study was a cross-sectional analysis of baseline data of our ongoing T2DM cohort. Patient selection, diagnosis, and clinical measurements have been described previously (21, 22). Briefly, from January 2018 to April 2020, 490 T2DM patients had been recruited from the Department of Endocrinology, Zhongshan Hospital (Xiamen), Fudan University (Xiamen, China). Among them, 241 patients without complete data on BMD measurements, hepatic ultrasonography scanning, or clinical measurements were excluded, and 249 T2DM patients (138 men and 111 women) were left in the present analysis. The inclusion criteria were as follows: (1) T2DM, (2) age ≥ 18 years, and (3) BMD testing and hepatic ultrasonography scanning measurements. The exclusion criteria were as follows: other types of diabetes (type 1 diabetes mellitus and secondary diabetes); severe liver and renal dysfunction; receiving or currently receiving estrogen and progesterone drugs, glucocorticoids, and calcium tablets; menopause by surgical intervention or at an unnatural age; familial fragility fracture; or unwillingness to participate in the study (21, 22). This study was approved by the Human Research Ethics Committee of the Zhongshan Hospital (Xiamen), Fudan University (Xiamen, China) (B2019-015). All patients provided written informed consent.

### 2.2 Measurements

Each patient received a face-to-face interview to collect sociodemographic data, lifestyle habits, present and previous health history, and medication utilization. Measurements of body weight, height, BMI (weight in kilograms divided by the square of the height in meters), waist circumference (WC), and arterial blood pressure (BP) were conducted as described previously (21, 22). Blood samples were collected in the morning after at least 12-h overnight fasting to measure fasting plasma glucose (FPG), glycosylated hemoglobin A1c (HbA1c), liver function, and lipid profiles, and were tested in the clinical laboratory of Zhongshan Hospital (Xiamen), Fudan University (Xiamen, China). Serum FPG, triglyceride (TG), total cholesterol (TC), high-density lipoprotein cholesterol (HDL-c), low-density lipoprotein cholesterol (LDL-c), aspartate aminotransferase (AST), alanine aminotransferase (ALT), and gamma-glutamyl transpeptidase (GGT) levels were measured using an analyzer (Roche Elecsys Insulin Test, Roche Diagnostics) as described previously (21–23). Homeostatic model assessment of insulin resistance (HOMA-IR) was used to estimate insulin resistance. Glycated hemoglobin (HbA1c) levels were determined using high-performance liquid chromatography (VARIANT II TURBO; Bio-Rad).

### 2.3 Hepatic steatosis and fibrosis indices and definition of MAFLD

Hepatic ultrasonography scanning was performed by an experienced radiologist using a GE LOGIQ P5 scanner (GE Healthcare, Milwaukee, USA) with a 4-MHz probe. Hepatic steatosis was diagnosed based on characteristic sonographic features, such as hepatorenal echo contrast, liver parenchymal brightness, deep beam attenuation, and vessel blurring (24). The fatty liver index (FLI) score was calculated based on the following formula: FLI = e^y^/(1+ e^y^) × 100, where y = 0.953 × ln (triglycerides, mg/dL) + 0.139 × BMI (kg/m^2^) + 0.718 × ln(GGT, U/L) + 0.053 × waist circumference (cm) – 15.745 (25). A cutoff FLI score of >60 was used to define hepatic steatosis in addition to the hepatic ultrasonography diagnosis (26). As an index of hepatic fibrosis, FIB-4 score was calculated using the formula: FIB-4 = age ([y] × AST [U/L])/((PLT [10^9^/L]) × (ALT [U/L])^1/2^), and a cutoff FIB-4 score >3.25 was defined as advanced hepatic fibrosis (27), which was also treated as one of the exclusion criteria in the present study. NAFLD fibrosis score (NFS) was calculated using the formula: NFS = −1.675 + 0.037 × age (years) + 0.094 × BMI (kg/m^2^) + 1.13 × impaired glucose tolerance or diabetes mellitus (yes = 1, no = 0) + 0.99 × (AST to ALT ratio) − 0.013 × platelet (10^9^/L) − 0.66 × albumin (g/dL) (28). Fatty liver was diagnosed by either hepatic ultrasonography diagnosis of hepatic steatosis or FLI score >60. Since all participants in the present study were diagnosed as T2DM, fatty liver patients with T2DM were defined as MAFLD based on the current international consensus definition for MAFLD (2, 3).

### 2.4 BMD measurement and definition of osteoporosis

Dual-energy x-ray absorptiometry (DXA) (QDR4500A, Hologic Inc., Waltham MA, USA) was used to measure BMD and operated by professional radiologists. BMD was checked at three different sites for each patient: total lumbar (L2–4), femur neck (FN), and total hip (TH), and was categorized into three groups according to the minimum T-score of BMD for each patient: normal (T ≥ −1.0), osteopenia (−2.5 < T < −1.0), and osteoporosis (T ≤ −2.5) (29).

### 2.5 Statistical analyses

Baseline data were presented as mean ± standard deviation (SD) for normally distributed continuous variables; median (interquartile range (IQR)) for non-normally distributed continuous variables or number and percentage for categorical variables. Differences between subjects stratified across MAFLD (yes vs. no) were tested using one-way analyses of variance (ANOVA) for normally distributed continuous variables, Wilcoxon rank-sum tests for non-normally distributed continuous variables, and the chi-square tests for categorical variables. Multivariable linear regression was analyzed to explore the associations of indices of hepatic steatosis [FLI score, fatty liver (FLI ≥ 60), fatty liver (hepatic ultrasonography scanning), and MAFLD] and hepatic fibrosis (FIB-4 score and NFS) with T-scores of BMD (L2-4, FN, TH, and the minimum) for all patients. Multivariable logistic regression analyses were conducted to calculate the adjusted odds ratios (ORs) and 95% confidence intervals (CIs) of hepatic steatosis and hepatic fibrosis indices for risks of osteopenia/osteoporosis (vs. normal) and osteoporosis (vs. osteopenia/normal) separately. Both multivariable linear regression and multivariable logistic regression analyses were adjusted for potential confounding variables in different models. In model 1, no confounding variable was adjusted. In model 2, age, sex, ever smoking and drinking, systolic blood pressure, diastolic blood pressure, diabetes duration, HbA1c, diabetes medical treatment, total cholesterol, triglycerides, HDL-C, and LDL-C were adjusted. In model 3, BMI as the index of obesity was further adjusted in addition to model 2. FLI, FIB-4 scores, and NFS were presented as per SD increase separately in the multivariable regression analyses. All *p*-values were two-sided and a *p*-value <0.05 was considered as statistical significance. All statistical analyses were conducted by using Stata14.0 (StataCorp, College Station, TX, USA).

## 3 Results

### 3.1 Demographic and clinical characteristics stratified across MAFLD

**Figure 1:**
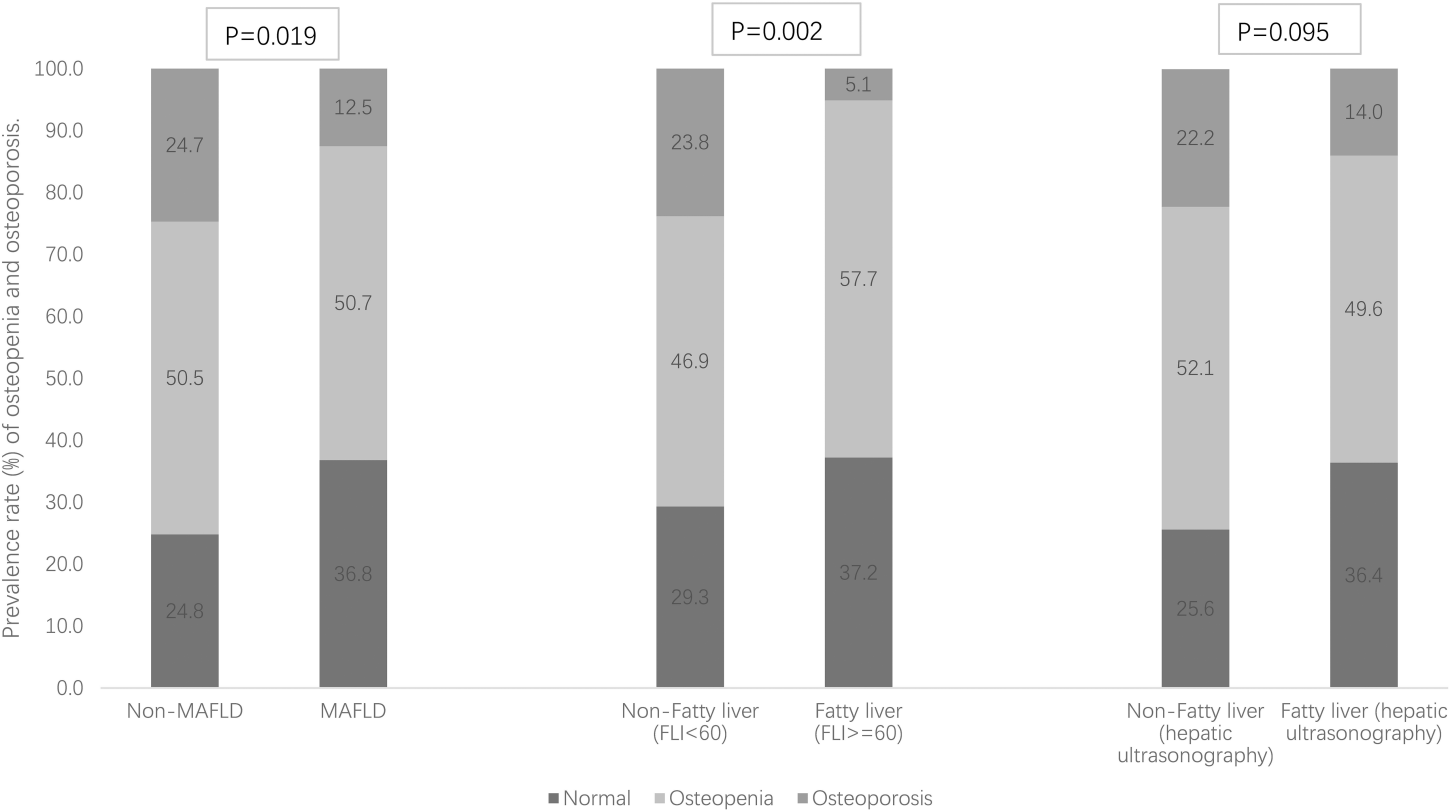
Prevalence rate (%) of osteopenia and osteoporosis stratified by MAFLD and fatty liver [defined as fatty liver index (FLI) ≥ 60 and hepatic ultrasonography separately].

Of the 249 T2DM patients, 138 were men and 111 were women with a mean ( ± SDs) age of 53.3 ± 12.3 and 60.6 ± 10.8 years and a diabetes duration median (IQR) of 5.0 (1.0–10.0) and 8.0 (3.0–13.0) years, respectively (both *p*-values < 0.05). Among them, 144 were identified as MAFLD, and the prevalence rates of MAFLD were 59.4%, 55.9%, and 57.8% for men, women and all, respectively. Differences of demographic and clinical characteristics stratified across MAFLD (yes vs. no) for all 249 patients are shown in **Table 1**. Patients with MAFLD showed significantly higher levels of body weight, BMI, waist circumference, diastolic BP, FPG, HOMA-IR, TG, AST, ALT, GGT, and CRP and significantly lower levels of age, diabetes duration, and HDL-c, compared to those without MAFLD. Additionally, MAFLD patients showed significantly higher indices of hepatic steatosis (FLI score and prevalence of fatty liver) but not hepatic fibrosis indices (FIB-4 score or NFS) than controls. MAFLD patients had significantly higher BMD T-scores (L2-4, FN, TH, and the minimum) than non-MAFLD patients (all *p*-values < 0.05). **Table 1** shows that the prevalence rates of osteopenia and osteoporosis in all 249 T2DM patients were 50.6% and 17.7%, respectively. **Figure 1** shows that the prevalence rate of osteoporosis was significantly lower in patients with MAFLD (12.5% vs. 24.7%) and patients with FLI ≥ 60 (5.1% vs. 23.8%) than controls, respectively (both *p*-values<0.05), but the prevalence rate of osteopenia and osteoporosis across fatty liver diagnosed by hepatic ultrasonography scanning was not statistically significant.

**Table 1:** Demographic, clinical characteristics, and bone mineral density stratified by MAFLD in 249 T2DM patients.

	MAFLD		Total	*p*-value
	No (*n* = 105)	Yes (*n* = 144)	(*N* = 249)	
Demographics and clinical characteristics				
Women (*n*, %)	49 (46.7)	62 (43.1)	111 (44.6)	0.571
Age (years)	58.8 ± 10.6	54.9 ± 13.0	56.6 ± 12.0	0.012*
Ever smoking (*n* (%))	36 (34.3)	47 (32.6)	83 (33.3)	0.785
Ever drinking (*n* (%))	19 (18.1)	41 (28.5)	60 (24.1)	0.059
Weight (kg)	60.5 ± 9.2	73.9 ± 11.8	68.3 ± 12.7	<0.001*
BMI (kg/m^2^)	22.5 ± 2.5	26.6 ± 3.8	25.1 ± 3.9	<0.001*
Waist (cm)	82.4 ± 7.4	93.1 ± 8.7	88.5 ± 9.7	<0.001*
Systolic blood pressure (mmHg)	129.5 ± 15.8	131.1 ± 16.2	130.4 ± 16.0	0.453
Diastolic blood pressure (mmHg)	79.9 ± 9.2	83.0 ± 10.4	81.7 ± 10.0	0.014*
Diabetes duration (years)	9.0 (2.0–12.0)	5.0 (1.0–10.0)	6.0 (1.0–11.0)	0.047*
Fasting plasma glucose (mmol/L)	7.74 ± 2.62	8.72 ± 3.18	8.30 ± 2.99	0.014*
HbA1c (%)	9.22 ± 2.46	9.17 ± 2.11	9.19 ± 2.26	0.851
HOMA-IR (*10^-6^mol*IU*L^−2^)	1.62 (1.24–2.85)	3.68 (2.62–4.93)	2.84(1.62–4.57)	<0.001*
Triglyceride (mmol/L)	1.18 (0.91–1.61)	2.03 (1.47–2.92)	1.61 (1.16–2.32)	<0.001*
Total cholesterol (mmol/L)	4.43 ± 1.12	4.55 ± 1.13	4.50 ± 1.12	0.384
HDL-cholesterol (mmol/L)	1.27 ± 0.35	1.02 ± 0.25	1.13 ± 0.32	<0.001*
LDL-cholesterol (mmol/L)	2.58 ± 1.02	2.51 ± 0.96	2.54 ± 0.99	0.617
AST (U/L)	16 (13–21)	18 (15–25)	17 (14–23)	0.005*
ALT (U/L)	18 (13–23)	23 (15–35)	20 (14–29)	<0.001*
GGT (U/L)	20 (15–26)	36 (23–49.5)	26 (19–42)	<0.001*
C-reactive protein (mg/L)	0.8 (0.5–1.7)	1.6 (0.8–3.3)	1.3 (0.7–2.8)	<0.001*
Diabetes treatment (*n* (%))				
Biguanides	52 (49.5)	79 (54.9)	131 (52.6)	0.405
Glycosidase inhibitor	35 (33.3)	26 (18.1)	61 (24.5)	0.006*
Sulfonylureas	35 (33.3)	47 (32.6)	82 (32.9)	0.908
TZD	7 (4.5)	26 (9.5)	33 (7.6)	0.059
Glinides	10 (9.5)	12 (8.3)	22 (8.8)	0.744
GLP-1 agonists	0 (0.0)	1 (0.7)	1 (0.4)	0.392
DPP-4 inhibitors	24 (22.9)	29 (20.1)	53 (21.3)	0.605
SGLT-2 inhibitors	1 (1.0)	1 (0.7)	2 (0.8)	0.822
Oral hypoglycemic medications use	80 (76.2)	108 (75.0)	188 (75.5)	0.829
Insulin use	43 (41.0)	34 (23.6)	77 (30.9)	0.003*
T-score of bone mineral density (BMD)				
Lumbar 2–4 T-score	−1.34 ± 1.39	−0.86 ± 1.20	−1.06 ± 1.30	0.004*
Femoral neck T-score	−1.47 ± 1.04	−1.09 ± 0.93	−1.25 ± 0.99	0.003*
Hip joint T-score	−1.10 ± 0.97	−0.65 ± 0.86	−0.84 ± 0.93	<0.001*
Minimum T-score	−1.84 ± 1.13	−1.39 ± 0.90	−1.58 ± 1.03	<0.001*
Categories of bone mineral density (BMD) (n (%))				0.019*
Normal (T-score ≥ −1.0)	26 (24.8)	53 (36.8)	79 (31.7)	
Osteopenia (−2.5 < T-score < −1.0)	53 (50.5)	73 (50.7)	126 (50.6)	
Osteoporosis (T-score ≤ −2.5)	26 (24.7)	18 (12.5)	44 (17.7)	
MAFLD indices				
FLI	21.1 ± 14.7	59.9 ± 22.0	43.1 ± 27.2	<0.001*
Fatty liver (FLI ≥ 60) (*N* (%))	0 (0.0)	78 (54.2)	78 (31.3)	<0.001*
Fatty liver (hepatic ultrasonography scanning) (*N* (%))	0 (0.0)	129 (89.6)	129 (51.8)	<0.001*
FIB-4 score	1.13 ± 0.50	1.11 ± 0.63	1.12 ± 0.58	0.762
NFS	−1.13 ± 1.09	−0.99 ± 1.14	−1.05 ± 1.12	0.337

*p < 0.05.

ALT, alanine transaminase; AST, aspartate transaminase; BMI, body mass index; DPP-4, dipeptidyl peptidase 4; FIB-4, Fibrosis-4; FLI, fatty liver index; GLP-1, glucagon-like peptide 1; GGT, gamma-glutamyl transpeptidase; HDL, high-density lipoprotein; HOMA-IR, homeostasis model assessment–insulin resistance; LDL, low-density lipoprotein cholesterol; MAFLD, metabolic associated fatty liver disease; NFS, NAFLD fibrosis score; SGLT-2, sodium glucose co-transporter 2; T2DM, type 2 diabetes mellitus; TZD, trazodone.

### 3.2 The associations of hepatic steatosis and fibrosis indices with T-score of BMD

The associations of hepatic steatosis [FLI score, fatty liver (FLI ≥ 60), fatty liver (hepatic ultrasonography scanning), and MAFLD] and hepatic fibrosis indices (FIB-4 score and NFS) with T-score of BMD in all the 249 T2DM patients were explored using multivariable linear regression analyses in three models with adjustment for different confounding variables. **Table 2** shows the regression coefficients (beta) with standard errors (SEs). In model 1 without adjustment, all hepatic steatosis indices, including FLI score, fatty liver (FLI ≥ 60), fatty liver (hepatic ultrasonography scanning), and MAFLD, were significantly and positively associated with all T-scores of BMD (L2-4, FN, TH, and the minimum) with the standardized regression coefficients (beta) ranging from 0.136 to 0.303 (all *p*-values<0.05). As for hepatic fibrosis indices, FIB-4 score, but not NFS, was significantly and negatively associated with all these T-scores with the beta value ranging from −0.196 to −0.142 (all *p*-values < 0.05). After adjusting for age, sex, ever smoking and drinking, systolic blood pressure, diastolic blood pressure, diabetes duration, HbA1c, diabetes medical treatment, total cholesterol, triglycerides, HDL-C, and LDL-C in model 2, FLI, fatty liver (FLI ≥ 60) and MAFLD were still positively associated with T-scores at TH and the minimum but not at L2–4, while fatty liver (hepatic ultrasonography scanning) and NFS, but not FIB-4 score, were significantly and positively associated with all T-scores. In model 3 with further adjustment for BMI (an index of general obesity) in addition to model 2, all associations of hepatic steatosis and fibrosis indices with T-score of BMD were not statistically significant.

**Table 2:** Multivariable adjusted linear regression coefficients of hepatic steatosis and fibrosis indices for BMD T-scores in 249 T2DM patients.

Sites	Model 1 ^†^			Model 2 ^‡^			Model 3 ^¶^		
	Beta	SE	*p*-value	Beta	SE	*p*-value	Beta	SE	*p*-value
Lumbar 2–4 T-score									
FLI (per SD increase)	0.184	0.083	0.004*	0.129	0.109	0.124	−0.047	0.173	0.723
Fatty liver (FLI ≥ 60, yes vs. no)	0.191	0.178	0.003*	0.144	0.211	0.057	0.070	0.253	0.442
Fatty liver (hepatic ultrasonography scanning, yes vs. no)	0.136	0.166	0.033*	0.082	0.187	0.253	0.033	0.194	0.659
MAFLD (yes vs. no)	0.180	0.165	0.004*	0.137	0.194	0.064	0.065	0.216	0.427
FIB-4 score (per SD increase)	−0.142	0.082	0.026*	−0.027	0.102	0.734	−0.033	0.101	0.668
NFS (per SD increase)	0.004	0.083	0.950	0.164	0.101	0.034*	0.105	0.108	0.203
Femoral neck T-score									
FLI (per SD increase)	0.260	0.062	<0.001*	0.253	0.078	0.001*	0.013	0.123	0.916
Fatty liver (FLI ≥ 60, yes vs. no)	0.242	0.134	<0.001*	0.195	0.152	0.007*	0.049	0.180	0.563
Fatty liver (hepatic ultrasonography scanning, yes vs. no)	0.163	0.126	0.010*	0.081	0.137	0.235	−0.006	0.138	0.928
MAFLD (yes vs. no)	0.191	0.126	0.003*	0.130	0.142	0.067	−0.009	0.154	0.909
FIB-4 score (per SD increase)	−0.195	0.062	0.002*	0.016	0.075	0.833	0.006	0.072	0.939
NFS (per SD increase)	−0.101	0.063	0.116	0.171	0.074	0.022*	0.062	0.077	0.425
Hip joint T-score									
FLI (per SD increase)	0.303	0.058	<0.001*	0.263	0.074	0.001*	−0.036	0.115	0.770
Fatty liver (FLI ≥ 60, yes vs. no)	0.290	0.124	<0.001*	0.222	0.143	0.002*	0.060	0.168	0.474
Fatty liver (hepatic ultrasonography scanning, yes vs. no)	0.213	0.117	0.001*	0.126	0.129	0.068	0.034	0.129	0.625
MAFLD (yes vs. no)	0.236	0.117	<0.001*	0.168	0.133	0.018*	0.023	0.144	0.769
FIB-4 score (per SD increase)	−0.182	0.059	<0.001*	0.010	0.071	0.892	−0.011	0.068	0.988
NFS (per SD increase)	−0.045	0.059	0.483	0.220	0.069	0.003*	0.105	0.072	0.174
Minimum T-score									
FLI (per SD increase)	0.244	0.065	<0.001*	0.192	0.082	0.016*	−0.041	0.129	0.740
Fatty liver (FLI ≥ 60, yes vs. no)	0.241	0.139	<0.001*	0.172	0.159	0.017*	0.053	0.189	0.534
Fatty liver (hepatic ultrasonography scanning, yes vs. no)	0.167	0.130	0.009*	0.090	0.142	0.192	0.018	0.145	0.796
MAFLD (yes vs. no)	0.214	0.123	0.001*	0.156	0.147	0.028*	0.049	0.161	0.533
FIB-4 score (per SD increase)	−0.196	0.064	0.002*	0.017	0.078	0.817	0.009	0.076	0.908
NFS (per SD increase)	−0.063	0.065	0.321	0.207	0.076	0.005*	0.122	0.080	0.118

*p < 0.05.

BMD, bone mineral density; FIB-4, fibrosis-4; FLI, fatty liver index; MAFLD, metabolic associated fatty liver disease; NFS, NAFLD fibrosis score; SE, standard error; SD, standard devation;T2DM, type 2 diabetes mellitus.

^†^ Unadjusted.

^‡^ Multivariable linear regression was adjusted for age, sex, ever smoking and drinking, systolic blood pressure, diastolic blood pressure, diabetes duration, HbA1c, diabetes medical treatment, total cholesterol, triglycerides, HDL-C, and LDL-C.

^¶^ Multivariable linear regression was adjusted for age, sex, ever smoking and drinking, systolic blood pressure, diastolic blood pressure, diabetes duration, HbA1c, diabetes medical treatment, total cholesterol, triglycerides, HDL-C, LDL-C, and BMI.

### 3.3 The associations of hepatic steatosis and fibrosis indices with risk of osteoporosis/osteopenia

Multivariable logistic regression analyses were conducted to test the associations of hepatic steatosis and fibrosis indices with risk of osteopenia/osteoporosis (vs. normal) in all the 249 T2DM patients with adjustment for different potential confounding variables in three models, and the adjusted ORs with 95% CIs are shown in **Table 3**. Without adjustment for any variable in model 1, MAFLD was significantly associated with decreased risk of osteopenia/osteoporosis, and the unadjusted OR (95% CI) was 0.565 (0.324–0.987, *p*-value = 0.045), while other hepatic steatosis and fibrosis indices, including FLI score, fatty liver (FLI ≥ 60), fatty liver (hepatic ultrasonography scanning), FIB-4 score, and NFS, were not significantly associated with risk of osteopenia/osteoporosis. In model 2 with adjustment for potential confounding variables similar to the multivariable linear regression analyses, FLI score, fatty liver (hepatic ultrasonography scanning), MAFLD, and NFS were significantly associated with decreased risk of osteopenia/osteoporosis, and the adjusted ORs (95% CIs) were 0.615 (0.416–0.909), 0.496 (0.253–0.975), 0.404 (0.200–0.817), and 0.539 (0.366–0.792) (all *p*-values < 0.05), respectively. With further adjustment for BMI in model 3 in addition to model 2, none of the hepatic steatosis and fibrosis indices was significantly associated with risk of osteopenia/osteoporosis.

**Table 3:** Unadjusted and adjusted odds ratios (ORs) of hepatic steatosis and fibrosis indices for risk of osteopenia/osteoporosis and osteoporosis in 249 T2DM patients.

Sites	Model 1 ^†^			Model 2 ^‡^			Model 3 ^¶^		
	OR	95% CI	*p*-value	OR	95% CI	*p*-value		95% CI	*p*-value
Osteopenia/osteoporosis vs. normal									
FLI (per SD increase)	0.794	0.605–1.042	0.096	0.615	0.416–0.909	0.015*	0.974	0.504–1.881	0.936
Fatty liver (FLI ≥ 60, yes vs. no)	0.699	0.396–1.235	0.218	0.565	0.269–1.185	0.131	1.173	0.464–2.967	0.735
Fatty liver (hepatic ultrasonography scanning, yes vs. no)	0.602	0.348–1.041	0.069	0.496	0.253–0.975	0.042*	0.676	0.329–1.386	0.285
MAFLD (yes vs. no)	0.565	0.324–0.987	0.045*	0.404	0.200–0.817	0.012*	0.636	0.286–1.416	0.268
FIB-4 score (per SD increase)	1.208	0.903–1.616	0.204	0.798	0.560–1.136	0.210	0.818	0.570–1.174	0.277
NFS (per SD increase)	0.993	0.760–1.297	0.957	0.539	0.366–0.792	0.002*	0.799	0.373–1.711	0.129
Osteoporosis vs. osteopenia/normal									
FLI (per SD increase)	0.536	0.366–0.784	0.001*	0.532	0.310–0.913	0.022*	0.849	0.354–2.037	0.715
Fatty liver (FLI ≥ 60, yes vs. no)	0.173	0.060–0.504	0.001*	0.171	0.049–0.596	0.006*	0.262	0.064–1.080	0.064
Fatty liver (hepatic ultrasonography scanning, yes vs. no)	0.568	0.293–1.100	0.093	0.689	0.311–1.525	0.358	1.113	0.448–2.766	0.817
MAFLD (yes vs. no)	0.434	0.224–0.843	0.014*	0.478	0.209–1.094	0.081	0.875	0.336–2.282	0.785
FIB-4 score (per SD increase)	1.446	1.080–1.936	0.013*	0.966	0.629–1.482	0.873	0.941	0.606–1.462	0.787
NFS (per SD increase)	1.062	0.766–1.474	0.717	0.631	0.404–0.986	0.043*	0.741	0.458–1.201	0.224

*p < 0.05.

FIB-4, fibrosis-4; FLI, fatty liver index; MAFLD, metabolic associated fatty liver disease; NFS, NAFLD fibrosis score; SD, standard devation;T2DM, type 2 diabetes mellitus.

^†^ Unadjusted.

^‡^ Multivariable linear regression was adjusted for age, sex, ever smoking and drinking, systolic blood pressure, diastolic blood pressure, diabetes duration, HbA1c, diabetes medical treatment, total cholesterol, triglycerides, HDL-C, and LDL-C.

^¶^ Multivariable linear regression was adjusted for age, sex, ever smoking and drinking, systolic blood pressure, diastolic blood pressure, diabetes duration, HbA1c, diabetes medical treatment, total cholesterol, triglycerides, HDL-C, LDL-C, and BMI.

### 3.4 The associations of hepatic steatosis and fibrosis indices with risk of osteoporosis

The associations of hepatic steatosis and fibrosis indices with risk of osteoporosis (vs. osteopenia/normal) were tested by using the multivariable logistic regression analyses in three models with adjustment for different potential confounding variables. In model 1 without adjustment for any variable, FLI score, fatty liver (FLI ≥ 60) and MAFLD were significantly associated with lower risk of osteoporosis, and the unadjusted ORs (95% CIs) were 0.536 (0.366–0.784), 0.173 (0.060–0.504), and 0.434 (0.224–0.843) (all *p*-values < 0.05), respectively. As for hepatic fibrosis indices, FIB-4 score, but not NFS, was significantly associated with an elevated risk of osteoporosis with an unadjusted OR (95% CI) of 1.446 (1.080–1.936, *p*-value = 0.013). In model 2 with adjustment for similar potential confounding variables to those of model 2 above, the adjusted ORs of FLI score, fatty liver (FLI ≥ 60), and NFS with risk of osteoporosis were statistically significant. However, with further adjustment for BMI in addition to model 2, none of the hepatic steatosis and fibrosis indices was significantly associated with risk of osteoporosis.

## 4 Discussion

With a total of 249 T2DM patients in the present study, we found that the prevalence rates of MAFLD, osteopenia, and osteoporosis were 57.8%, 50.6%, and 17.7%, respectively. Patients with MAFLD showed significantly higher BMD T-scores and lower prevalence rates of osteopenia/osteoporosis and osteoporosis than those without MAFLD. All hepatic steatosis indices were positively associated with BMD T-scores, while FIB-4 score was negatively associated with BMD T-scores; MAFLD was significantly associated with decreased risk of osteopenia/osteoporosis and osteoporosis. After adjusting for all the potential confounding variables, especially the general obesity index of BMI, in either multivariable linear regression analyses or multivariable logistic regression analyses, we found that none of the hepatic steatosis and hepatic fibrosis indices was independently associated with BMD T-scores or risks of osteopenia/osteoporosis or osteoporosis.

Existing evidence on the association between NAFLD and osteoporosis focused on general populations in a few observational studies with inconsistent results. Ciardullo et al. conducted cross-sectional analyses on the association between NAFLD and osteoporosis based on the data from NHANES 2017–2018 for participants older than 50 years, and they found that liver steatosis was associated with lower prevalence of osteoporosis. After adjusting for potential confounders, they found that liver steatosis was not associated with osteopenia or osteoporosis in the US population aged 50 years old and above (17). Xie et al. found a negative relationship between NAFLD and lumbar BMD using NHANES 2017–2018 data for populations aged 20 to 59 years. However, the association between NAFLD and osteoporosis turned out to be insignificant with adjustment for BMI (30). Li et al. performed a cross-sectional study in 2,031 participants over 50 years old in NHANES 2017–2018 and found the positive association of MAFLD with BMD as well as the negative association of MAFLD with femoral osteoporosis. However, the association of MAFLD with osteoporosis became insignificant when BMI was adjusted as a potential confounding factor (18). Liu et al. recently used the same data of NHANES 2017–2018 for those aged 50 or above and found that patients with MAFLD had a higher BMD and a lower risk of osteoporosis than those without MAFLD, but the multiple logistic regression models showed that participants with MAFLD had no increased risks of osteoporosis after adjusting for confounding variables (19). To the best of our knowledge, we were probably the first to report the associations of liver steatosis with BMD and the risk of osteopenia/osteoporosis for T2DM patients. In the present cross-sectional analyses of 249 T2DM patients, we found that hepatic steatosis indices were positively associated with BMD T-scores and that MAFLD was significantly associated with decreased risk of osteopenia/osteoporosis and osteoporosis. However, all these associations became statistically non-significant with adjustment for the potential confounding factors, especially including BMI as the index of general obesity.

Evidence about the association of hepatic fibrosis or cirrhosis with osteoporosis was limited and controversial. A meta-analysis of six case–control studies in 2016 concluded that, despite the high heterogeneity among these studies, patients with cirrhosis showed increased prevalence of osteoporosis, and suggested more accurate screening of BMD in patients with liver cirrhosis for adequate osteoporosis management (31). Abdominal ultrasonography for diagnosis of NAFLD and liver fibrosis or cirrhosis is often subjective and lacks sensitivity, especially for obese subjects, and a non-invasive and quantitative evaluation method has been widely used for assessment of fatty liver or liver fibrosis nowadays (25). In a cross-sectional study of 129 subjects with NAFLD assessed using transient elastography, Kim et al. found that liver fibrosis was independently associated with lower BMD and elevated risk of osteopenia and osteoporosis in NAFLD subjects (32). Li used data from NHANES 2017–2018 and found a positive association between liver stiffness and BMD as well as a negative association of liver fibrosis with femoral osteoporosis, but all these associations became statistically non-significant after adjusting for BMI and other confounding factors (18). Similarly, Ciardullo et al. found that liver fibrosis was not associated with osteopenia or osteoporosis in the US population older than 50 years (17). However, in a cross-sectional study from 1,872 obese individuals in Rome, Italy, Barchetta et al. found that higher FIB-4 score, an index of liver fibrosis, was independently associated with lower BMD and increased risk of osteopenia/osteoporosis (20). Moreover, available evidence about the associations of liver fibrosis with BMD and the risk of osteopenia/osteoporosis for T2DM patients was quite limited. Based on two cohorts containing 46 subjects with biopsy-proven NAFLD and 445 subjects with proton magnetic resonance spectrum-proven NAFLD, Zhu et al. found that NAFLD-associated hepatic fibrosis was negatively associated with BMD (33). However, it should be noted that all subjects in that study were postmenopausal women with NAFLD and T2DM or impaired glucose regulation simultaneously. In our present study, the associations of hepatic fibrosis with BMD and the risk of osteopenia/osteoporosis for T2DM patients were explored for the first time. Our data revealed that increasing FIB-4 score, but not NFS, was negatively associated with BMD T-score at L2–4, FN, TH, and the minimum, and was positively associated with elevated risk of osteoporosis (vs. osteopenia/normal). However, in the multivariable regression analyses with adjustment for the potential confounding factors, especially including BMI, all these associations were statistically non-significant. Therefore, our findings were quite consistent with evidence from NHANES 2017–2018 that hepatic fibrosis was not independently associated with either BMD or risk of osteopenia/osteoporosis.

The pathophysiology mechanisms linking the associations of liver steatosis and fibrosis with BMD and risk of osteopenia/osteoporosis have not been well established. Previous studies revealed that numerous pathogenic mediators, including IGF-1, fibronectin, the RANKL/OPG system, and several cytokines, have important roles in the pathogenesis of bone loss in chronic liver disease (34). The linkage between hepatic inflammation/fibrosis and adipose inflammation and insulin resistance has been established via the release of a cluster of inflammatory mediators from adipose tissue, for example, TNF-α, IL-6, and monocyte chemoattractant protein-1 (35). The development of osteopenia, resulting from the systemic inflammation and insulin resistance observed in this disorder, could at least partly explain the association between BMD and liver fibrosis. Besides the pro-inflammatory states in T2DM patients that could lead to both hepatic steatosis and fibrosis and osteoporosis, the microbiota of the intestinal tract has been considered to be closely linked to bone (36), and the gut–liver–bone axis might be critical in the associations of osteopenia and/or osteoporosis with MAFLD according to its regulatory effect on both resorption and formation process of bone (37). In the present study, we found that liver steatosis and fibrosis were not significantly associated with BMD and risk of osteopenia/osteoporosis independent of obesity. One possible reason was that all our study subjects were T2DM patients with higher levels of obesity, insulin resistance, lipotoxicity, and reactive oxidative stress than the general population, which may therefore prevent us from finding the independent associations of liver steatosis and fibrosis with BMD and risk of osteopenia/osteoporosis in T2DM patients. Importantly, a recent systemic review summarized that the preserved or even increased BMD as well as bone fragility with consequent increased susceptibility to fracture is common in T2DM patients compared to that of control, in which multiple regulatory mechanisms including microvascular disease, advanced glycation end products, osteoprotegerin/receptor-activator of nuclear factor κB ligand, the Wnt/β-catenin pathway, osteonectin, and fibroblast growth factor 23 are likely involved (14). Collectively, the complicated underlying mechanisms of hepatic steatosis and fibrosis on regulation of BMD could be helpful for understanding insights into osteopenia/osteoporosis, partially in T2DM patients. However, future studies on the potential mechanisms linking these associations are still warranted.

Existing evidence on the relationship of MAFLD/NAFLD and hepatic fibrosis with osteopenia and/or osteoporosis was mainly seen in the general population, while few studies have focused on T2DM patients, although the prevalence of T2DM is increasing rapidly worldwide. To the best of our knowledge, our study was the first study to explore the associations of MAFLD and hepatic fibrosis indices with BMD and risk of osteopenia and/or osteoporosis in T2DM patients. However, it should be acknowledged that our findings had the following limitations. Firstly, our present findings were from cross-sectional analyses of baseline information of our ongoing T2DM cohort, and we could not determine the temporal sequences of the associations of MAFLD and hepatic fibrosis indices with BMD and risk of osteopenia and/or osteoporosis. Secondly, all the 249 T2DM patients were recruited from a hospital from Xiamen, China; therefore, selection bias in the present study was obvious and their representativeness was quite limited. Thirdly, owing to the small sample size of our study, we could not extrapolate the present findings to other populations due to our limited power. Last but not the least, we did not have data on more rigorous assessments of liver fibrosis, such as liver Fibroscan test; thus, we could only use indices of serum biomarkers on liver fibrosis in the present study. Therefore, future studies with larger sample sizes and more rigorous assessments of hepatic steatosis and fibrosis, particularly those based on prospective cohort study designs, are needed.

The present study showed that the prevalence of MAFLD, osteopenia, and osteoporosis was quite high in T2DM patients. Hepatic steatosis indices, including MAFLD, were positively associated with BMD T-scores, while hepatic fibrosis index (FIB-4 score but not NFS) was negatively associated with BMD T-scores; MAFLD was significantly associated with lower risk of osteopenia/osteoporosis and osteoporosis. However, with adjustment for obesity, hepatic steatosis and fibrosis indices were not independently associated with either BMD or risk of osteopenia/osteoporosis or osteoporosis in T2DM patients. Nevertheless, screening of MAFLD, hepatic fibrosis, BMD, and osteopenia/osteoporosis is important for T2DM patients, especially from the perspective of fracture prevention.

## Data availability statement

The raw data supporting the conclusions of this article will be made available by the authors, without undue reservation.

## Ethics statement

The studies involving humans were approved by Human Research Ethics Committee of Zhongshan Hospital (Xiamen, China), Fudan University. The studies were conducted in accordance with the local legislation and institutional requirements. The participants provided their written informed consent to participate in this study.

## Author contributions

WZ: Conceptualization, Data curation, Funding acquisition, Writing – original draft, Writing – review & editing. YL: Data curation, Formal Analysis, Writing – review & editing. SL: Investigation, Methodology, Validation, Writing – review & editing. JZ: Data curation, Validation, Writing – review & editing. KW: Data curation, Validation, Writing – review & editing. ZL: Conceptualization, Data curation, Formal Analysis, Investigation, Project administration, Validation, Writing – original draft. NC: Conceptualization, Funding acquisition, Supervision, Writing – review & editing. XC: Conceptualization, Funding acquisition, Supervision, Writing – review & editing.
